# Exploring Weight Loss Medication Discourse: Mixed Methods Analysis of US-Based Facebook Posts

**DOI:** 10.2196/89732

**Published:** 2026-05-07

**Authors:** Elizabeth Dennard, Katrina Makres, Amrutha Alibilli, Vidur Jain, Anne Wang, Roger Abim-Karmon, Shaniece Criss, Yulin Hswen, Quynh C Nguyen, Alexandria Ratzki-Leewing, Rozalina G McCoy, Thu T Nguyen

**Affiliations:** 1 Deparment of Epidemiology and Biostatistics, School of Public Health, University of Maryland, College Park, 4254 Stadium Drive, College Park, MD, 20742, United States; 2 Furman University, Greenville, SC, United States; 3 National Institutes of Health, Bethesda, MD, United States; 4 University of Maryland School of Medicine, Baltimore, MD, United States; 5 University of Maryland Institute for Health Computing, North Bethesda, MD, United States; 6 Division of Endocrinology, Diabetes, and Nutrition, University of Maryland School of Medicine, Baltimore, MD, United States

**Keywords:** weight loss, obesity, digital health, internet, research

## Abstract

**Background:**

Despite the documented clinical efficacy of weight loss medications, few large-scale mixed methods studies have captured the experiences of individuals taking these medications.

**Objective:**

This study aims to examine dominant themes and public narratives about the perceived benefits, challenges, identity-based experiences, and the broader sociocultural framing of glucagon-like peptide-1 receptor agonist use on Facebook.

**Methods:**

We used CrowdTangle to collect 2500 US-based Facebook posts from January 1, 2022, to May 31, 2024. Bidirectional encoder representations from transformers topic modeling was used to identify latent topics, followed by a qualitative thematic analysis.

**Results:**

An analysis of 2500 Facebook posts revealed distinct thematic patterns across five unique subcategories: (1) healthy weight management, (2) weight loss medications, (3) discussion specific to semaglutide, (4) discussions specific to Ozempic, and (5) public figures and media. Thirteen overarching themes emerged through this thematic analysis, with the most common themes including weight management programs (n=702), neutral content related to weight loss medication use (n=466), harms of weight loss medication use (n=373), benefits of weight loss medication use (n=329), and access to these medications (n=188).

**Conclusions:**

Social media provides critical insights into individual experiences with pharmacotherapy and weight management. Enhanced public education may optimize the safe use of glucagon-like peptide-1 receptor agonists for weight loss.

## Introduction

In the United States, more than one-third of adults are currently affected by obesity [1], a figure projected to approach one-half by 2030 [2]. Obesity is a major driver of type 2 diabetes, cardiovascular disease, and other leading causes of death in the United States [3]. Glucagon-like peptide-1 receptor agonists (GLP-1 RAs) have emerged as a highly effective pharmacologic approach to treat obesity and type 2 diabetes. These agents mimic the incretin hormone glucagon-like peptide-1 (GLP-1) to stimulate insulin secretion, delay gastric emptying, and suppress appetite. Multiple large-scale randomized trials, including STEP, SUSTAIN, and SURMOUNT [4,5], have demonstrated clinically meaningful weight loss and cardiometabolic benefits in individuals with obesity [6,7]. In response, regulatory approvals have expanded, and demand has surged; recent estimates suggest that 1 in 8 US adults has used a GLP-1 RA, with 40% citing weight loss as their primary goal [8].

Even though more than 45% of US adults meet eligibility criteria for GLP-1 RA use, uptake remains uneven due to limitations posed by high costs, insurance restrictions, and the still evolving evidence on long-term outcomes [9]. Discontinuation is common and often followed by weight regain, especially in the absence of sustained lifestyle support [10]. Therapeutic plateaus, unrealistic expectations, and adverse events may further undermine adherence. Frequently reported adverse events include gastrointestinal symptoms—nausea, vomiting, abdominal pain, constipation, and/or diarrhea—often experienced within the first month of GLP-1 RA use and during dose escalation [11]. Other less frequently assessed adverse events of GLP-1 RA use among people with overweight or obesity include gastroesophageal reflux disease, gastroenteritis, pancreatitis, and gallbladder-related outcomes (cholelithiasis and cholecystitis) [12]. Overall, current findings support the widespread use of GLP-1 RAs to treat obesity, diabetes, and other cardiovascular-kidney-metabolic disorders, while also highlighting frequently reported adverse events that require further investigation to inform patient counseling and adverse effect mitigation strategies.

Moreover, the cultural framing of these medications has shifted rapidly, with celebrity endorsements and esthetic motivations fueling widespread media attention and off-label use [13,14]. Deeper insight into real-world lived experiences and expectations of GLP-1 RA users is essential for informing equitable, person-centered treatment and education strategies. While clinical trials and guideline developments have centered on efficacy and safety, much less research has been conducted on how these medications are understood outside formal health care settings. Recently, social media platforms have become rich venues for user-driven health narratives—providing diverse perspectives that remain largely unexplored in the GLP-1 RA literature.

Previous studies have leveraged social media data to examine discourse surrounding pharmaceutical advertising and user-reported behavioral experiences [15,16]. However, findings from these studies focused on advertising and specific user experiences, leaving broader patterns of GLP-1 RA discourse unexplored. Analyzing this discourse at scale requires tools capable of preserving narrative nuance while uncovering broader thematic patterns. This study leverages bidirectional encoder representations from transformers topic (BERTopic), a transformer-based topic modeling approach, paired with qualitative content analysis to examine public narratives about GLP-1 RA use on Facebook. By applying this mixed method strategy to a large corpus of user-generated posts, we aimed to elucidate dominant themes related to perceived benefits and harms, access challenges, identity-based experiences, and the broader sociocultural framing of these medications.

## Methods

### Data Source

Over time, Facebook has evolved into one of the most globally used platforms, attracting over 3 billion annual users [17]. Approximately 71% of US adults report using the platform with disproportionately higher usage among older adults and women compared to other platforms [18]. This pattern aligns with GLP-1 use among women who are more likely to be currently taking a GLP-1 RA [19]. Facebook posts were collected using CrowdTangle, a publicly available tool that enables systematic exploration of content across social media platforms [20]. CrowdTangle provides data from public accounts (7 million Facebook pages, groups, and verified profiles) and allows users to refine results using parameters such as keywords, timeframes, and geolocation [20]. Currently available tools for researchers include Meta Content Library and Content Library API [21]. For this study, data were collected using keyword-based filters, yielding information such as post content, image URLs, captions, dates of posts, and engagement metrics.

### Data Preparation

Data from English-language posts originating from US-based users were collected between January 1, 2022, and May 31, 2024. GLP-1 RA use has surged since 2022. Tirzepatide was approved by the US Food and Drug Administration (FDA) for diabetes management in May 2022 and was quickly used for off-label obesity management before receiving FDA approval in June 2023 [22-24]. To examine the rise in GLP-1 RA discussions online, we used data collected through CrowdTangle from January 2022 to May 2024; CrowdTangle was discontinued in August 2024 [21]. The keyword list (Table 1) was developed based on a combination of prior literature and clinical expertise. A total of 64,202 posts were retrieved. Text preprocessing included the removal of symbols, hyperlinks, HTML tags, and other special characters. Key terms were identified using regular expression methods.

**Table 1.** List of keywords used for capturing posts from Meta.

ID	Keywords
1	Weight loss drugs
2	Ozempic
3	Weight loss medication
4	Weight loss treatments
5	Weight management
6	Mounjaro
7	Tirzepatide
8	Weight loss medications
9	Miracle drug
10	Miracle weight loss
11	Miracle jab
12	Ozempy
13	Semaglutide
14	Wegovy
15	GLP-1^a^
16	GLP-1 agonist
17	GLP-1s
18	Weight management drugs
19	Weight management meds
20	Weight management medications
21	Skinny pen
22	Skinny jab
23	Skinny jots
24	Weight loss shot
25	Miracle diet drug
26	Liraglutide
27	Saxenda
28	Victoza

a GLP-1: glucagon-like peptide-1.

### Quantitative Analysis

To identify key topics across Facebook posts, we applied BERTopic, a modern topic modeling approach that uses advanced language models to group similar content based on meaning [25]. Unlike traditional techniques such as latent Dirichlet allocation, which rely on word frequency patterns, BERTopic captures deeper contextual relationships between words, allowing for more meaningful and nuanced topic discovery [26]. The process began by converting each post into a numerical representation of its meaning using a pretrained language model. These representations were then simplified using uniform manifold approximation and projection (UMAP), which helps organize complex data. Next, a clustering algorithm known as hierarchical density-based spatial clustering of applications with noise grouped the posts into clusters based on similarity [27,28]. To interpret each topic, we used a modified version of term frequency–inverse document frequency (TF-IDF) called class-based TF-IDF, which highlights the most distinctive words within each group [25]. This technique improves the clarity of each topic and sets BERTopic apart from other topic modeling tools.

BERTopic was implemented in Python (Python Software Foundation) using the BERTopic library, with default settings for dimensionality reduction and clustering [25]. Several exploratory runs were conducted by adjusting parameters to evaluate the stability and interpretability of the results. Four parameters in total were adjusted: UMAP dimensionality reduction parameters (n_neighbors, n_components) and BERTopic parameters (nr_topics, min_topic_size). In the final model, we used n_neighbors=13, n_components=9, nr_topics=5, and min_topic_size=100. The final model was selected based on semantic coherence and the clarity of topic keywords. Following deduplication, the dataset consisted of 50,013 unique Facebook posts. These topics provided an initial thematic structure that helped organize the large Facebook dataset and informed the subsequent qualitative content analysis.

### Qualitative Content Analysis

A thematic analysis was conducted on a random sample of 500 Facebook posts for each of the 5 topics identified using BERTopic, for a total of 2500 posts. Thematic analysis is a widely used qualitative method that allows for the analysis of free-response data using an established codebook [29]. First, the authors implemented an inductive approach by reviewing 20 posts from each subcategory and discussing emerging themes to establish an understanding of the dataset through familiarization [29]. Then, the authors constructed themes through pattern formation and identification, assessed thematic diversity, and tested the codebook’s operational abilities. The final codebook comprised 13 overarching themes, each containing unique subthemes (Table 2). Once the codebook was finalized, 2 authors independently conducted postscreening of the 2500 posts. Conflicts were resolved through discussion between the two reviewers until consensus was reached based on detailed justifications. The finalized themes and subthemes were then applied to guide a comprehensive thematic analysis of the sample, offering insight into the range of topics discussed in public discourse surrounding GLP-1 RA medications on Facebook.

**Table 2.** Codebook for thematic analysis.

Codebook ID	Themes	Subthemes
1	Natural and whole foods	Multiple foods: health outcome Multiple foods: nonhealth outcome Protein: health outcome Protein: nonhealth outcome Fiber: health outcome Fiber: nonhealth outcome Whole grains: health outcome Whole grains: nonhealth outcome Vegetables: health outcome Vegetables: nonhealth outcome Oils: health outcome Oils: nonhealth outcome Other: health outcome Other: nonhealth outcome
2	Physical activity	Health outcome Nonhealth outcome
3	Vegetarian or vegan	Health outcome Nonhealth outcome
4	Supplements	Health outcome Nonhealth outcome
5	Weight loss medications—promotion	Weight loss Health benefits Both weight loss and health benefits Celebrities
	Weight loss medications—neutral	Education Updates on regulations
6	Weight loss medications—harms	Harming health Exploiting insecurities Too much weight loss Temporary results of weight loss medications Celebrities
7	Speculation about weight loss medication use	N/A^a^
8	Preference for unmedicated weight loss	N/A
9	Access to weight loss medications	Availability of medication Cost Insurance Lawsuits and investigations
10	Weight management programs	Selling or promoting weight management programs Advice
12	Hormone therapy	N/A
13	Research studies	Weight loss Health benefits Both weight loss and health benefits
14	Business expansion (products capitalizing on the rise in use of weight loss medications)	N/A
99	Not relevant	N/A

a N/A: not applicable.

### Ethical Considerations

This study did not involve human participants and was conducted in accordance with ethical standards determined by the University of Maryland College Park Institutional Review Board (2072551‐1). Given the nature of the study, examination of publicly available social media posts did not involve direct interaction or communication with human subjects. Additionally, posts were anonymized to uphold user privacy.

## Results

### Overview

BERTopic was used to analyze 50,013 posts referencing GLP-RAs and initially explored several configurations. A target topic size of 6 with a minimum topic size of 100 posts was chosen to prevent overly granular clusters. One topic was automatically flagged as a low-density outlier, yielding a total of five topics. The 5-topic model was then selected based on the clarity of cluster separation visualized in the intertopic distance map (Multimedia Appendix 1), which is a 2D UMAP projection of topic embeddings representing relative semantic distances between topics. There was no overlap between the 5 topics, and comparable distances were demonstrated, supporting the validity of the model.

**Figure 1.**
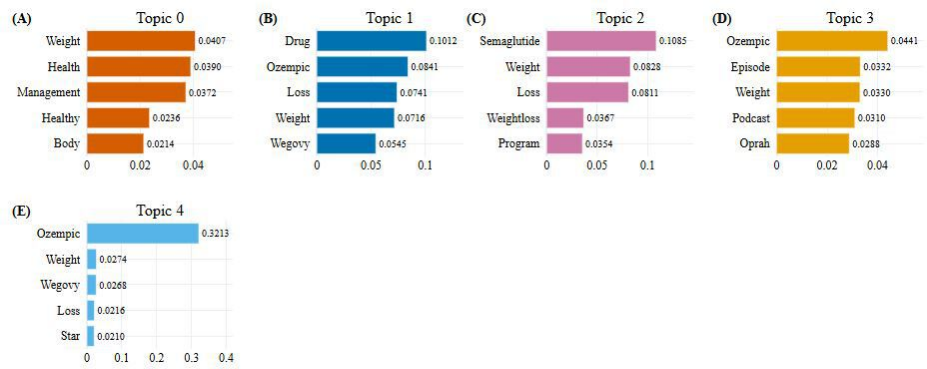
Topic word scores. This figure illustrates the top 5 keywords for each topic (A-E) based on class-based term frequency–inverse document frequency scores. These terms represent the most representative words within each topic.

Topics were categorized using the top keywords identified using class-based TF-IDF scores (Figure 1). BERTopic modeling yielded 5 distinct topics: healthy weight management, weight loss medications, semaglutide, public figures, and Ozempic. These broad topics serve as a foundation for understanding the dominant narratives surrounding weight loss drugs on social media. They provide a structured lens to interpret public discourse, identify recurring patterns, and uncover underlying concerns and motivations.

Topic 0 (healthy weight management) emerged as a prominent theme characterized by keywords such as “weight,” “health,” and “management.” Posts in this category focused on lifestyle-based approaches to combating obesity. Topic 1 (weight loss medications) captured broader discussions about pharmaceutical interventions, featuring user experiences, medication comparisons, and commentary on side effects. Topic 2, characterized by the keyword “semaglutide,” reflected in-depth discourse on the clinical use and effectiveness of this specific medication. Topic 3 (public figures) centered on the role of celebrities and influencers in shaping perceptions of weight loss treatments. Finally, topic 4 (Ozempic) focused on its off-label use for weight loss, concerns around access and affordability, and growing public interest.

Then, a content analysis was conducted on a random sample of 2500 Facebook posts. As depicted in Table 2, 13 distinct themes emerged during the qualitative analysis, each comprising various subthemes. The most popular themes across the sample included weight management programs, neutral weight loss medication posts, harms of weight loss medications, promotion of weight loss medications, and access to weight loss medications. Table 3 provides a detailed overview of each theme and subtheme, accompanied by illustrative quotations to highlight specific examples of Facebook user statements. A total of 89 posts were excluded from the final analysis because they lacked substantive references to weight management or the population of interest.

**Table 3.** Examples of weight management posts by theme and subtheme.

Themes and subthemes	Post
Natural and whole foods and supplements	
Multiple foods	“When it comes to maintaining a healthy and balanced diet, it’s important to remember the significance of incorporating a variety of nutrient-rich foods. One excellent addition to your daily intake is nuts and berries. These small yet powerful ingredients are packed with essential vitamins, minerals, and antioxidants that offer numerous health benefits...Incorporating nuts and berries into your daily diet can enhance your overall nutritional intake and contribute to a healthier lifestyle. So, remember to include them in your meals, snacks, or even as toppings for yogurt, cereal or however you like!”
Protein	“Top fat loss nutrition tip I wish I knew earlier: get 30‐40 grams protein at every meal Why? Glad you asked top 5 reasons to eat high protein foods. Muscle growth: protein is essential for building and repairing muscles, which is crucial for overall strength and fitness. Weight management: high-protein diets can help you feel full for longer periods, reducing overall calorie intake and supporting weight loss or maintenance. Metabolism boost: protein has a higher thermic effect compared to fats or carbs, meaning your body burns more calories digesting it, potentially aiding in weight management...”
Fiber	“Trying to lose weight? Fiber is your friend! High-fiber foods can help to promote a feeling of fullness and satiety by delaying digestion and adding bulk to meals. For instance, starches like chickpeas (found in hummus) may help with weight management. Try it with some pita slices and fresh veggies. It’s delicious!”
Whole grains	“Is ’oat-zempic’ a substitute for Ozempic? Experts say this trend does not mimic the way these drugs used for weight loss work and could have negative health impacts.”
Vegetables	“Eating fruits and vegetables offers a myriad of health benefits. Packed with essential nutrients, fiber, and vitamins, they support overall well-being. Studies link them to a lower risk of chronic diseases, improved weight management, and better digestive health. Share your favorite fruits or veggies!”
Other	“Hydrating doesn’t have to be boring! Spice up your water with refreshing mint, orange, and cucumber for the perfect summer drink that will keep you healthy and energized!”
Supplements	“Spirulina is a nutrient-dense blue-green algae that offers several health benefits. Here are some key advantages of incorporating spirulina into your diet: Rich in Nutrients Spirulina is a powerhouse of nutrients, including protein, vitamins (B-complex vitamins, especially B12), minerals (iron, calcium, magnesium), and essential fatty acids. High Protein Content It is an excellent source of plant-based protein, making it a valuable addition to vegetarian and vegan diets. Antioxidant Properties Spirulina contains antioxidants like phycocyanin, which helps protect cells from damage caused by free radicals. Weight Management The protein and nutrient content in spirulina may contribute to a feeling of fullness, potentially aiding in weight management. Blood Sugar Control Preliminary research indicates that spirulina may have a positive effect on blood sugar levels, making it potentially beneficial for individuals with diabetes or those at risk...”
Physical activity	
Health outcome	“Elevate Your Health with Cardio! Burn calories, boost mood, and strengthen your heart. From weight management to increased endurance, these workouts have got your back (and heart) covered!”
No health outcome	“Beta-hydroxybutyrate, or BHB, is a key ingredient in SiselTHIN. BHB’s are naturally occurring salts found in the body during ketosis. Getting into ketosis can be difficult to achieve through diet alone. Exogenous (provided externally) BHB’s can provide the critical fuel needed during a keto diet and will help you get into ketosis faster and stay there so you can achieve your weight management goals. SiselTHIN is part of our January promo pack. Have you gotten yours yet? Get your packs and save while the deal is still available.”
Diet (vegan or vegetarian)	
Health outcome	“Did you know plant-based diets can contribute to weight management and overall well-being? This Lent, embark on a journey of self-discovery with BombayFoodJunkies. Discover a world of flavorful and healthy vegan options on our menu! Ready to try plant-based food for 40 days? Book a table now and enjoy yummy, guilt-free meals!”
Weight loss medications—promotion	
Weight loss	“Unlock the secret to feeling full and satisfied! Discover the hormone that signals to your brain that your stomach is full, leading to decreased hunger and increased feelings of satisfaction. This hormone, called GLP-1, can even help your body use more energy from stored fat.”
Health benefits	“Ozempic protects kidneys, boosts survival in diabetes patients with chronic kidney disease.”
Both	“Drugs like Wegovy and Ozempic have demonstrated success in treating obesity and diabetes. Can they treat depression too?”
Celebrities	“Whoopi Goldberg revealed she used weight loss drug Mounjaro to help her shed some pounds after weighing nearly 300 pounds in late 2021.”
Weight loss medications—neutral	
Education	“Here’s what psychiatrist Allison Young, MD, says patients should be asking their doctors if they think new prescription weight loss drugs might help with weight gain due from antidepressants or other drugs prescribed for mental illness.”
Regulatory	“Based on the new, unprecedented results, Eli Lilly announced plans to seek fast-track approval from the U.S. Food and Drug Administration to advertise and sell the drug for weight management.”
Weight loss medications—harms	
Harming health	“Ozempic weight loss has patients needing to get rid of sagging skin. The primary reason for this new challenge with sagging skin is the loss of skeletal muscle.”
Exploiting insecurities	“In the first few months of 2023, it seems like society has taken some serious steps backward when it comes to our relationship with our bodies. There are new headlines about Ozempic and weight loss nearly every day, and many of us are still reeling from the American Academy of Pediatrics’ January recommendation that kids as young as 12 be prescribed weight loss drugs (and that those as young as 13 be prescribed surgery)... In our latest article, we talked with experts about what parents can say and do to help their kids build resilience and push back against the idea that we should all be striving to be thinner. Give it a read for actionable advice on protecting your kiddo from today’s rampant fatphobia”
Too much weight loss	“A great supplement to consider if you are experiencing rapid weight loss due to certain medications. Ask one of the Rocky Mountain Pharmacystaff. They’ll fill you in on the details.”
Temporary results	“Sun Kim cautions against using popular weight-loss drugs as a quick fix: “We have to think of obesity as a chronic disease,” she explains. "Someone with high blood pressure doesn’t expect to stop taking blood pressure medications once normal blood pressure has been achieved. If you stop taking semaglutide, you generally regain the weight back.”
Celebrities	“Lizzo fans criticize her for taking Ozempic to lose weight.”
Access to weight loss medications	
Availability	“For months, multiple diabetes drugs like Ozempic have been in high demand with short supply.”
Cost	“Wegovy’s high price and the huge increase in people taking it companies reconsidering when and how to reimburse use of such treatments to prevent a steep spike in health insurance costs.”
Insurance	“America’s screwed-up healthcare system is preventing millions from receiving a new “life-saving” weight-loss drugs like Wegovy and Ozempic.”
Lawsuits and investigations	“Federal prosecutors accuse 36-year-old Isis Navarro Reyes of using the social media platform to illegally sell bacteria-contaminated prescription medications, including Ozempic, that she had obtained from Central and South America.”
Weight management programs	
Selling or promoting programs	“If you currently use Saxenda or Ozempic contact us for more information or to apply! If you participated in a Saxenda or Ozempic study with us in the last 6 months, you will not be able to participate in this session. Thank you for understanding.”
Advice	“Most of us want to eat better. But how should you start? Here’s a helpful article that offers some simple ways to develop a healthier diet without depriving yourself and explains how working with a nutritionist can help support your weight loss or weight management goals.”
Research studies or statistics	
Weight loss	“In this trial, adolescents with obesity assigned to weekly subcutaneous semaglutide plus lifestyle intervention had a greater reduction in BMI than those who received lifestyle intervention alone.”
Health benefits	“Novo Nordisk’s Ozempic slows diabetic kidney disease progression in trial”
Both	“A late-stage trial of Eli Lilly’s weight loss drug Zepbound showed promising results in treating sleep apnea symptoms in obese adults, the company announced Wednesday, the latest findings suggesting popular weight loss drugs have the potential to treat conditions other than obesity or type 2 diabetes.”
Business expansion	
None	“Rapid weight loss can be for your body confidence, but it can lead to some unexpected changes in your facial appearance. We’re here to help!”
Hormone therapy	
None	“Hormone harmony: boost estrogen levels and balance hormones PCOS Support: Aid is reducing symptoms commonly related with polycystic ovary syndrome such as irregular menstrual cycle Weight Management: Chromium has been shown to regulate and support blood sugar levels and in turn help in fat loss Skin & Hair Strength: L-Glutathione is an antioxidant that can help reduce wrinkles and improve complexion with added biotin for hair and nail health (Click here to order”
Speculation	
None	“Curious about Ashley Graham’s stunning weight loss journey? Wondering if it was through a dedicated diet plan, the buzzed-about Ozempic, or perhaps weight loss surgery? Dive into the details and discover the truth behind her transformation. Learn more:”
Preference for unmedicated weight loss	
None	“You don’t need to manage your weight. Your body is already doing this for you. Anyone selling weight management as a strategy for health promotion is selling a product that will result in an eventual decline of physical or mental health or both. I appreciate that you might want to attempt to manage your body. I understand that this is a product sold, repeatedly. In different packages and as various plans. Please save your money. The body cant discern weight management strategies from famine. No matter how fancy the package, it is not fancy business. And it is only about business. ($$$) Not yours...What are you doing this weekend? *my page is educational and does not represent nutrition therapy or medical care.”

### Quantitative Content Analysis of the 5 Topics

**Figure 2.**
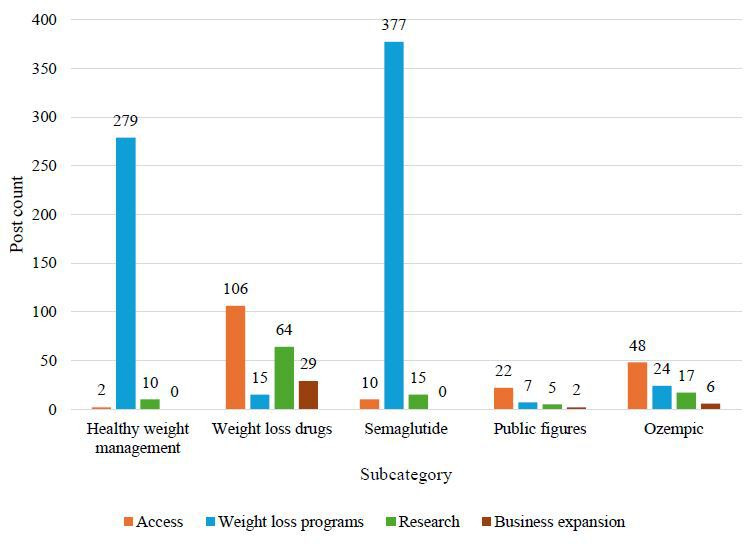
Weight management posts by subcategory and theme (group 1: access, weight loss programs, research, and business expansion). This bar graph illustrates the total number of weight management posts across subcategories (x-axis) and themes (represented by the colors of the bars). The x-axis categorizes posts by theme (access, weight loss programs, research, and business expansion) and subcategory (healthy weight management, weight loss medications, semaglutide, public figures, and Ozempic), while the y-axis displays the corresponding post counts.

**Figure 3.**
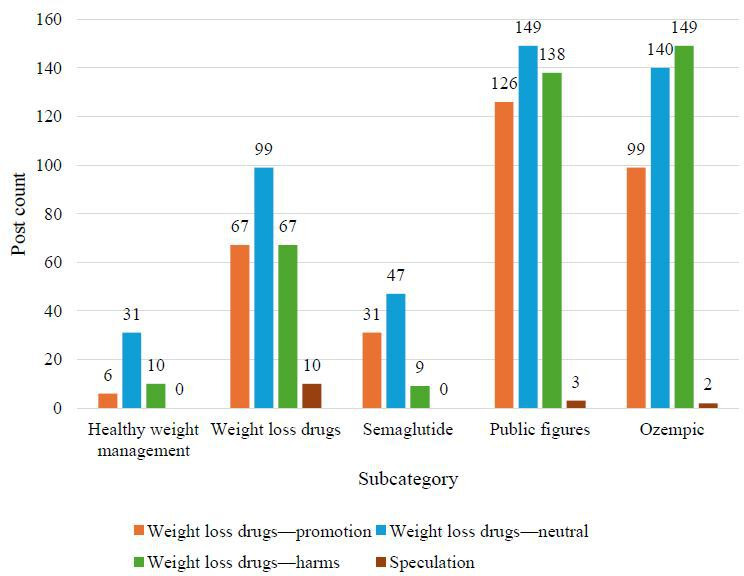
Weight management posts by subcategory and theme (group 2: weight loss medications—promotion, weight loss medications—neutral, weight loss medications—harms, and speculation). This bar graph illustrates the total number of weight management posts across subcategories (x-axis) and themes (represented by the colors of the bars). The x-axis categorizes posts by theme (weight loss medication promotion, neutral, harms, and speculation of weight loss medication use) and subcategory, while the y-axis displays the corresponding post counts.

**Figure 4.**
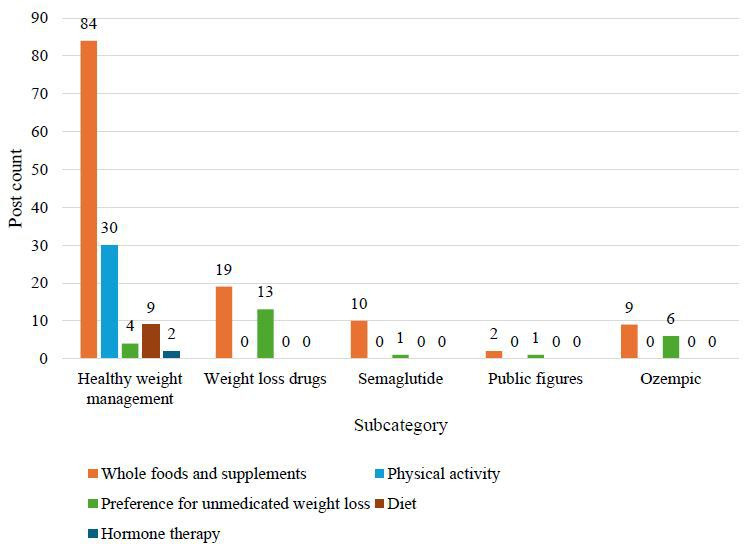
Weight management posts by subcategory and theme (group 3: natural and whole foods and supplements, physical activity, preference for unmedicated weight loss, diet, and hormone therapy). This bar graph illustrates the total number of weight management posts across subcategories (x-axis) and themes (represented by the colors of the bars). The x-axis further categorizes posts by theme (natural and whole foods and supplements, physical activity, diet, preference for unmedicated weight loss, and hormone therapy), while the y-axis provides the corresponding post counts.

The five BERTopic-derived topics included (1) healthy weight management, (2) weight loss medications, (3) semaglutide, (4) public figures and media, and (5) Ozempic. Across the 5 topics, several overarching themes emerged, such as natural and whole foods and supplements, physical activity, vegan or vegetarian diets, attitudes toward weight loss medications, speculation about medication use, preference for nonpharmacologic options, access to weight loss medications, weight loss programs, commercial expansion, hormone therapy, and research studies. Additionally, post counts for each theme are described across the 5 BERTopic-derived topics (Figures 2-4). The 5 most prevalent themes were as follows: “weight management programs,” “weight loss medications—neutral,” “weight loss medications—harms,” “weight loss medications—promotion,” and “access to weight loss medications.” “Weight management programs” referred primarily to program promotion, supplement sales, and company advertisements. “Weight loss medications—neutral” included informational content and regulatory updates. “Weight loss medications—harms” referred to negative aspects such as adverse events and exploitative messaging. “Weight loss medications—promotion” focused on perceived benefits, and “access to weight loss medications” included issues related to cost, insurance, and availability. Illustrative examples of posts for each theme and subtheme are presented in Table 3.

### Healthy Weight Management Subcategory

The most common themes in this group were “weight management programs,” including 279 (55.8%) posts; “natural and whole foods and supplements,” including 84 (17%) posts; and “weight loss medications—neutral,” including 31 (6%) posts. Most “weight management programs” posts (n=245) promoted programs, while fewer (n=34) offered health advice. Most posts referenced health outcomes, such as diabetes, obesity, and cardiovascular disease. “Weight loss medications—neutral” posts included information, expert opinions, or federal guidance on weight loss medications. This category also included notable references to physical activity (n=30).

### Weight Loss Medications Subcategory

In this subcategory, the most common themes included “access to weight loss medications,” comprising 106 (21.2%) posts; “weight loss medications—neutral,” comprising 99 (20%) posts; “weight loss medications—promotion,” comprising 67 (13%) posts; and “weight loss medications—harms,” comprising 67 (13.4%) posts. Access posts referenced availability (n=46), cost (n=15), insurance coverage (n=26), and lawsuits or investigations (n=19). Neutral posts included educational content, with fewer posts mapping to the “updates on regulations” subtheme. Promotional posts highlighted weight loss (n=30), health benefits (n=9), both weight loss and health benefits (n=18), and celebrity posts (n=10), allowing for further discussion regarding the weight loss benefits of these medications. With identical theme counts, posts focused on harms raised concerns about health risks, exploiting insecurities, excessive weight loss, temporary results, and celebrity posts.

### Semaglutide Subcategory

The distribution of posts was dominated by “weight management programs,” including 377 (75.4%) posts, followed by “weight loss medications—neutral,” including 47 (9%) posts and “promotion,” including 31 (6%) posts. Most posts related to this topic promoted paid or supplement-based weight management programs (n=373), consistent with the commercial focus observed across the full dataset. Neutral posts covered educational content and webinar information (n=45) or regulatory updates (n=2), while promotional posts centered around weight loss success (n=23) and weight loss combined with health benefits (n=7).

### Public Figures and Media Subcategory

This subcategory involved weight loss medication posts, with “neutral” accounting for 149 (29.8%) posts, “harms” accounting for 138 (27.6%) posts, and “promotion” comprising 126 (25.2%) posts. “Neutral” posts included educational facts or webinar information (n=148) and FDA regulation updates (n=1). Harm-related posts frequently referenced celebrities (n=114). Some discussed how weight loss medications harm one’s health (n=18), while others noted the exploitation of insecurities (n=6). This category also included posts in which celebrities were critical of weight loss medications or public complaints regarding celebrity weight loss medication usage. Promotional posts highlighted celebrities (n=115), including reports of positive results. Additional subthemes from positive posts included weight loss (n=6), health benefits with weight loss (n=3), and general health benefits (n=2). There was an almost even divide of posts about celebrities in the harm and promotion themes regarding weight loss medications. The public figures and media subcategory had the highest number of posts surrounding celebrities, with a total of 229 posts.

### Ozempic Subcategory

The most common themes were “weight loss medications—harms,” including 149 (29.8%) posts; “neutral,” including 140 (28%) posts; and “promotion,” including 99 (20%) posts. Harms of weight loss medications included adverse events (n=51), exploiting insecurities (n=5), excessive weight loss (n=1), temporary results (n=2), and celebrity posts (n=90). The discussion of the negative impacts of Ozempic on Facebook was more common than that of its positive effects. The majority of neutral weight loss medication posts were linked to education (n=134), with only a few mapping to the regulatory subtheme (n=6). For positive posts linked to weight loss medications, celebrity posts were identified most frequently, while posts highlighting weight loss, health benefits, or both appeared less frequently. Unlike other subcategories, Ozempic included the most posts linked to the harms of weight loss medications (n=149).

### Themes Across the Data Sample: Descriptions and Example Quotes

#### Weight Management Programs

Posts under this theme frequently promoted weight management programs, advertised weight loss medications, and shared general advice. These posts often included persuasive sales messages:

*Transform your journey, sculpt your dreams!! Introducing the ultimate duo: Emsculpt and semaglutide/Tirzepatide For EXTREME fast major and amazing results!! Elevate your results and redefine your contours and step into a confidence that’s uniquely yours. Contact us/text us to learn more.*

Conversely, other posts from established programs were more neutral and often informative, offering general advice on nutrition, exercise, and supplements while supporting consumer weight management journeys: *“*Nutrition tip: choose whole foods over processed ones for a nutrient-rich diet that supports your weight management goals.*”*

#### Weight Loss Medications—Neutral

These posts shared educational information about GLP-1 RAs. From expert webinars to FDA updates, the neutral posts advanced factual knowledge about weight loss medications: *“*FDA approves weight-loss drug Wegovy to reduce heart-disease risks.*”*

#### Weight Loss Medications—Harms

Various posts highlighted the negative physical and psychological consequences of using weight loss medications. Common physical adverse health outcomes included gastrointestinal distress:

*Ozempic and Wegovy can cause gastrointestinal issues such as constipation and nausea. Can increasing your fiber intake help?*

In addition, long-term weight loss efficacy was questioned by users discussing excessive or temporary weight loss: *“*Although Ozempic and Wegovy have been found to help persons with obesity lose weight, they are not necessarily a foolproof approach for long-term weight loss and weight management.*”* Common psychological harms included exploiting consumer insecurities by targeting body image, social comparison, and conformity. Additionally, increased media coverage and celebrity endorsements of weight loss medications may heighten the pressure on consumers to conform to Western beauty standards and perpetuate fatphobia.

#### Weight Loss Medications—Promotion

Users commonly discussed weight loss and other health benefits associated with GLP-1 RA use. Various posts highlighted the duality of both weight loss and improved holistic health outcomes:

*23lbs down and starting month 3 this week!! Compounded Semaglutide I feel INCREDIBLE! My autoimmune issues are at an all time low! My skin is clearer. And I had to buy NEW clothes!!!*

### Access to Weight Loss Medications

The final most prevalent theme included access to weight loss medications, which encompassed GLP-1 RA availability, affordability, lawsuits, and investigations. Many users were concerned about GLP-1 RA shortages: *“*Can’t get my Ozempic and am out. Anyone know which pharmacy has the 2 in stock?*”* Furthermore, the increasing costs presented another accessibility barrier: *“*Zepbound has a much lower sticker price than its rival, Novo Nordisk’s Wegovy.” Additionally, insurance coverage was described across numerous posts: *“*Some lawmakers say the United States cannot afford to keep a decades-old law that prohibits Medicare from paying for new weight loss drugs.*”* Finally, lawsuits and investigations often discussed regulatory pricing and other guidelines that may impact public access to the medications: *“*Bernie Sanders called a vote to subpoena the chief of Novo Nordisk’s U.S. division over pricing of Ozempic and Wegovy.*”*

Overall, social media discussions about weight management revealed a complex landscape with a myriad of themes and a wide spectrum of public sentiment. Social media users shared their weight loss successes and perceived health benefits, as well as adverse physical and psychological events they experienced. The data also revealed exposure to marketing, including a variety of promotional programs and advertisements. Public figures and celebrities were commonly featured in messaging related to endorsements and critical discourse on weight loss medications. Finally, posts that discussed medication access underscored significant concerns about GLP-1 RA shortages, high costs, limited insurance coverage, and ongoing lawsuits that could impact weight loss medication pricing and availability.

## Discussion

### Principal Findings

Mixed methods analysis of Facebook posts discussing GLP-1 RA medications revealed 13 distinct thematic patterns across 5 unique topics. The most prevalent themes included weight management programs, weight loss medications, and access to weight loss medications. Healthy weight management posts primarily featured weight loss programs, natural and whole foods, supplements, and physical activity. In the weight loss medications subcategory, access and educational posts were most frequently identified. Semaglutide-related posts were dominated by program promotions, while public figures and media primarily focused on weight loss medications, with a high percentage of celebrity influence in both harm and promotional contexts. Ozempic posts most frequently discussed harms and education, with fewer posts promoting the use of weight loss medications. Finally, online discourse was heavily shaped by commercial interests, personal experiences, and celebrity influence, highlighting a need for evidence-based messaging around weight loss interventions.

Our findings highlight novel themes across identified topics while also confirming current sentiments regarding weight loss medications among social media users. Facebook users often shared personal experiences, motivations for GLP-1 RA use, and research supporting the use of weight loss medications. These sentiments align with existing studies that describe the benefits of GLP-1 RA use for sustainable weight loss [30]. In addition to physical health benefits, studies have documented the mental health benefits of weight loss medications. A recent systematic review reported associations between GLP-1 RAs and reductions in depressive symptoms, suicidal ideation, alcohol use, substance use, and binge eating behaviors [31]. However, some adverse psychiatric outcomes have been documented in pharmacovigilance analyses, with varying effects across different types of weight loss medications [31].

Beyond clinical outcomes, prior research highlights the stigma surrounding GLP-1 agonists, rooted in cultural ideals favoring “disciplined” weight loss—an image embedded in fatphobic ideologies [13]. High costs, limited availability, and lack of insurance coverage exacerbate class-based fatphobia, disproportionately affecting lower-income individuals with fewer options for pharmacological treatment [13]. Scholars emphasize that sustainable cultural change requires initiatives beyond medical interventions, including public health campaigns and educational resources that foster body diversity, empathy, and an understanding of the complexity of obesity as a multifaceted health condition [13].

The widespread promotion of GLP-1 RAs has been heavily influenced by celebrity culture where the pressure to maintain one’s physical appearance and weight is critical. Weight management culture in the United States is entangled with long-standing trends of fatphobia, body image pressures, and evolving medical approaches to obesity, as evidenced by increased usage of GLP-1 RAs [13]. Celebrities’ early adoption of GLP-1 RAs amplifies the desirability of these medications. High-profile celebrities like Oprah Winfrey have acknowledged their use of weight loss medications, while others are widely suspected of doing the same [32]. The growing visibility of celebrity use of weight loss medications can further influence public attitudes toward obesity, fatphobia, and body image [13].

### Strengths and Limitations

By analyzing 2500 randomly selected Facebook posts, this study offers insights into public perceptions and information-sharing practices surrounding weight loss medications. By analyzing diverse subcategories, we captured a wide range of content, including educational, commercial, and celebrity influences. This study addresses existing data gaps, such as social media discourse and the cultural framing of GLP-1 RA medications, which are relevant to public health communication, patient education, and policy development. Additionally, this study employed BERTopic, a modeling technique that captures contextual and semantic relationships in text and is well suited to informal social media discourse. Compared to traditional models, BERTopic enhances topic coherence, dynamic reduction, and outlier detection. After removing duplicates and outliers, we randomly sampled 2500 posts for detailed analysis, ensuring a diverse dataset spanning educational content, personal experiences, and commercial promotion.

However, several limitations merit attention. First, the analysis was limited to Facebook and may not reflect discussions occurring on other social media platforms. Additionally, Crowdtangle data did not provide information on the type of accounts that originated from the included Facebook posts. Second, while the keyword list was informed by prior literature and author expertise, it was not exhaustive, potentially missing relevant online discussions. Third, we lacked demographic and engagement metrics (eg, likes and shares), which could have offered additional insights for interpreting influence. Finally, the analysis was restricted to English-language posts, limiting generalizability to non-English-speaking communities.

### Conclusions

This study underscores the complexity of social media discourse surrounding GLP-1 RAs and the intersection of commercial, clinical, and cultural narratives. As weight loss medications gain increasing popularity across diverse populations, it is imperative to ensure that their use is not only effective but also safe. This mixed methods study highlights the depth and complexity of social media discourse surrounding GLP-1 RAs as an interweaving narrative of commercial, clinical, and cultural perspectives. Our analysis reveals that GLP-1 RA use is shaped by individual and social contexts, including personal values, lived experiences, expectations, and stigma. Future studies should build upon this descriptive and exploratory study to examine long-term patterns of GLP-1 RA use, benefits, and side effects across diverse populations. Additionally, future studies can consider social media platform diversity and how social media users navigate sources of evidence-based information to counteract the spread of misinformation online. Research is also needed to support understanding of user experiences with non-FDA-approved products (such as compounded drugs) or medications obtained outside of traditional health care settings (such as directly from the manufacturer), as data on adverse effects would not be fully captured in electronic health records or claims. Our analysis lays important groundwork for future work focused on understanding GLP-1RAs through a more complete, person-centered lens—a critical component of evidence-based medicine.

## Supplementary material

10.2196/89732 Multimedia Appendix 1 Intertopic distance map of topic embeddings generated by BERTopic with circles topic proportional to size.

## Abbreviations

BERTopic: bidirectional encoder representations from transformers topic
FDA: Food and Drug Administration
GLP-1: glucagon-like peptide-1
GLP-1 RA: glucagon-like peptide-1 receptor agonist
TF-IDF: term frequency–inverse document frequency
UMAP: uniform manifold approximation and projection
