# Supplementary figures and images for: Exploring Weight Loss Medication Discourse: Mixed Methods Analysis of US-Based Facebook Posts
Source: JMIR Infodemiology. 2026 May 7;6:e89732. doi: 10.2196/89732 (PMC13153451; doi:10.2196/89732)

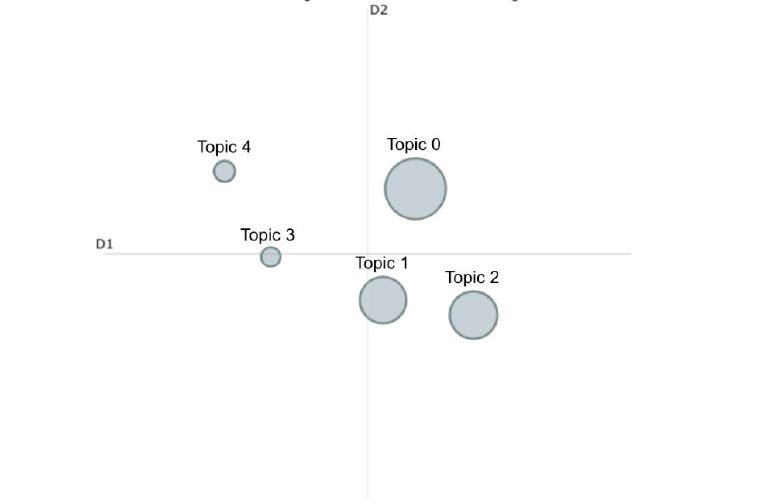

Supplement: Multimedia Appendix 1 [file infodemiology-v6-e89732-s001.jpg]
